# Evaluation of the efficacy and safety of either or both mirabegron and silodosin, as a medical expulsive therapy for distal ureteric stones

**DOI:** 10.1007/s11255-023-03880-y

**Published:** 2023-12-02

**Authors:** Mohammad Sayed Abdel-Kader, Ahmed Mohammad Sayed, Sondos Mohammad Sayed, Mostafa AbdelRazek

**Affiliations:** 1 Urology Department, Faculty of Medicine, South Valley University, Qena, Egypt; 2 Faculty of Medicine, South Valley University, Qena, Egypt

**Keywords:** Mirabegron, Silodosin, Expulsive, Stone

## Abstract

**Objective:**

To evaluate efficacy and safety of either or both silodosin and mirabegron as MET for distal ureteric stones ≤ 10 mm.

**Patients and methods:**

This study enrolled a total of 105 patients, aged between 20 and 56 years, diagnosed by single radiopaque distal ureteral stone measuring ≤ 10 mm. The recruitment period spanned from May 2020 to December 2021. The patients were randomly divided into three groups, with each group consisting of 35 participants. Group A received a once-daily dose of 8 mg of silodosin, group B received a once-daily dose of 50 mg of mirabegron, and group C received a combination of both medications. Treatment was administered to all patients until the stone was expelled or for a maximum duration of four weeks. The stone-free rate was determined by analyzing KUB films with or without ultrasonography.

**Results:**

The rate of stone expulsion was significantly higher in group C compared to groups A and B (P = 0.04 and P = 0.004, respectively). The mean (standard deviation) time for stone expulsion in groups A, B, and C was 14 ± 2.3 days, 11 ± 3.1 days, and 7 ± 2.2 days, respectively. Group C demonstrated a significantly shorter stone expulsion time compared to groups A and B (P = 0.001 and P = 0.04, respectively). The frequency of renal colic in group C was significantly lower than that in groups A and B, resulting in a reduced requirement for analgesics (P < 0.05). Anejaculation occurred at a significantly higher rate in the silodosin group (73.9%) and combination group (84%) compared to the mirabegron group (P < 0.05).

**Conclusions:**

The findings of this study suggest that both silodosin and mirabegron are effective treatments for the expulsion of lower ureteric stones. Furthermore, the combination of these medications leads to an increased rate of stone expulsion and a reduced duration of expulsion.

## Introduction

Approximately 20% of urinary stones are located in the ureter, with 70% of them being in the distal ureter. While most stones pass spontaneously, they can cause acute pain (renal colic), creating a need for effective pain relief and improved stone passage methods [1].

The spontaneous expulsion rate of distal ureteric stones is between 25 and 53% for stones ≤ 10 mm in size, making conservative treatment an option for such cases [2]. The spontaneous passage of ureteric stones depends on various factors, including stone size, shape, location, ureteral muscle spasm, ureteral wall edema, and anatomical abnormalities [3].

Medical expulsive therapy (MET) is commonly used to facilitate expulsion of ureteric stones. Alpha-blockers are frequently employed and have been proven effective in clinical practice. Silodosin, a highly selective α1A-adrenoceptor antagonist, has been used as a MET for distal ureteric stones by inducing relaxation of the ureteral muscles and achieving higher stone expulsion rates than other blockers [4].

However, alpha-blockers can have adverse effects due to their mechanism of action, including anejaculation, nausea, dizziness, and orthostatic hypotension. Thus, there is a need for novel, more effective agents with lower complication rates [5].

Beta-3 adrenoceptors (B3AR) are widely present in ureteral smooth muscles, urothelium, and interstitial cells, mediating adrenergic stimulation for ureteral relaxation [6]. Mirabegron, a selective B3AR agonist, has been introduced as a novel MET and offers an effective and safe alternative to previous MET agents that act via different pathways [7].

In this study, we aimed to assess the efficacy and safety of silodosin, mirabegron, or a combination of both as MET for distal ureteric stones ≤ 10 mm.

## Patients and methods

This prospective, randomized study was conducted between May 2020 and December 2021. Ethical committee approval was obtained prior to the study. The inclusion criteria consisted of patients over 18 years old with a single radio-opaque stone ≤ 10 mm located in the distal ureter. Exclusion criteria included single functioning kidney, impaired renal function, multiple or bilateral and radiolucent ureteric stones, severe persistent pain, urinary tract infection, severe hydronephrosis (grade IV according to *SFU grading system*), uncontrolled hypertension, pregnancy, anatomical abnormalities, current use of alpha-blockers, and previous ureteral surgery.

**Fig. 1:**
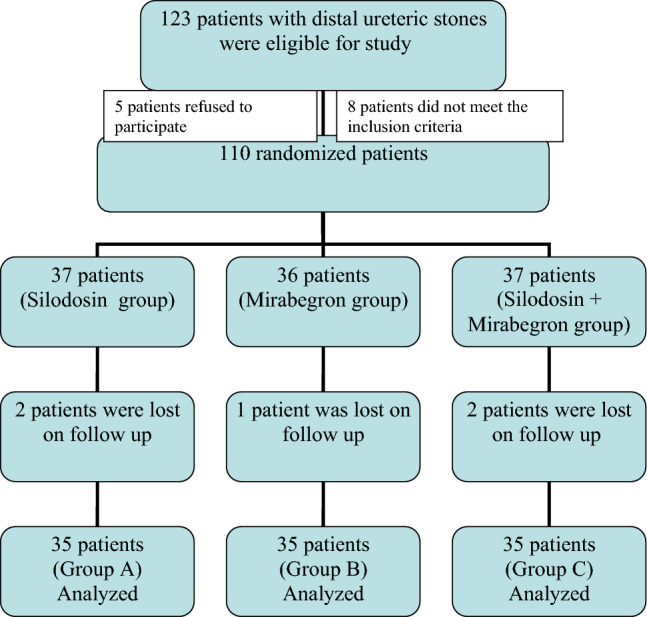
Flow chart of the study

A total of 123 patients with distal ureteric stones were initially assessed for eligibility to participate in the study. Out of these, 18 patients were excluded for various reasons (as shown in Fig. 1). Ultimately, 105 patients who met the inclusion criteria were enrolled in the study. Informed written consent was obtained from all patients after providing them with an explanation of the study procedures and possible side effects of the drugs.

Sample size calculated using G*Power software, the significance level (alpha) α value is 0.05, which corresponds to a 5% chance of making a Type I error (rejecting the null hypothesis when it is true). We Determine the statistical power 1—beta (β). The statistical power is the probability of correctly rejecting the null hypothesis when it is false used value for statistical power is 80%.

The patients were randomly divided into three equal groups using a computer-generated random number table prepared by a statistician who was not part of the research team. Each group consisted of 35 patients. Group A was administered a daily dose of 8 mg of silodosin, Group B received a daily dose of 50 mg of mirabegron, and Group C received a combination of 8 mg of silodosin and 50 mg of mirabegron. The treatment was given to all patients until either the expulsion of the stone occurred or a maximum of 4 weeks had passed.

Patients underwent various assessments, including medical history collection, general examination, blood urea and serum creatinine tests, urine analysis, urine culture, kidney, ureter, and bladder X-ray (KUB), and non-contrast CT. Additionally, patients were advised to take a 30 mg tablet of ketorolac orally during pain attacks, along with the assigned medication. All patients were instructed to consume a minimum of 2500–3000 mL of water daily and strain their urine to detect any potential stones.

Follow-up evaluations were conducted weekly at the outpatient clinic. These evaluations involved collecting information on stone passage, the number of episodes of renal colic, the total amount of extra analgesia required, and any adverse effects associated with the administered drugs. Ultrasonography was also performed during the follow-up visits. The follow-up continued until the stone passed spontaneously or until the treatment was discontinued after 4 weeks. The stone-free rate (SFR), indicating the absence of any stones, was determined using KUB film with or without ultrasonography on a weekly basis. Treatment failure was defined as the persistence of stones confirmed by radiological examination after 4 weeks.

The primary objective of the study was to determine the rate of stone expulsion, while the secondary objectives included assessing the time it took for the stones to be expelled, the number of pain attacks, the amount of additional analgesia required, and the adverse effects associated with the medication. The collected data was reported, reviewed, coded, and entered into the Statistical Package for Social Science (SPSS) version 23. The comparison between the groups was performed using the chi-square test, with a p-value of less than 0.05 considered statistically significant.

Data analysis was performed using the Statistical Package for Social Science (SPSS) version 23. Statistical comparisons between groups were conducted using the chi-square test, with P < 0.05 considered significant.

## Results

The study included 105 out of 123 patients, and Table 1 presents the demographic and clinical characteristics. There were no statistically significant differences between the groups in patient age, sex, BMI, stone size, laterality, and hydronephrosis degree (P > 0.05), as shown in Table 1. Additionally, there were no statistically significant differences between the groups in terms of pre- or post-treatment serum creatinine levels.

**Table 1:** Comparison between groups according to demographic data and clinical characterestics

Variables	Group A No. = 35	Group B No. = 35	Group C No. = 35	*P* value
Age mean ± SD	36.65 ± 6.81	38.62 ± 7.88	37.81 ± 8.11	0.615
Sex (%)				0.213
Male	23 (65.71%)	22 (62.86)	25 (71.43%)	
Female	12 (34.29%)	13 (37.14%)	10 (28.57%)	
Body mass index (Kg/m^2^) mean ± SD	24.35 ± 2.22	23.6 ± 2.93	24.15 ± 2.6	0.334
Laterality (%)				0.876
Right	24 (68.57%)	22 (62.86%)	23 (65.71%)	
Left	11 (31.43%)	13 (37.14%)	12 (34.29%)	
Stone size (mm) mean ± SD	7.44 ± 1.32	7.11 ± 1.25	7.54 ± 1.25	0.348
Hydronephrosis degree (%)				0.716
Non or Mild	14 (40%)	16 (45.71%)	15 (42.86%)	
Moderate	21 (60%)	19 (54.29%)	20 (57.14%)	
Pretreatment creatinine	1.1	1.06	1.15	0.864

Stone expulsion was reported in 20 out of 35 patients (57.1%) in Group A, 18 out of 35 patients (51.4%) in Group B, and 33 out of 35 patients (94.3%) in Group C. The stone expulsion rate was significantly higher in Group C compared to Groups A and B (P = 0.04 and P = 0.004, respectively). The mean (SD) expulsion time in Groups A, B, and C was 14 ± 2.3 days, 11 ± 3.1 days, and 7 ± 2.2 days, respectively. The stone expulsion time was significantly shorter in the combination group (Group C) compared to the silodosin (Group A) and mirabegron (Group B) groups (P = 0.001 and P = 0.04, respectively).

Regarding renal colic episodes, the combination group (Group C) had a significantly lower frequency in comparison to silodosin (Group A) and mirabegron (Group B) groups, and less analgesic medication was required (P < 0.05), as shown in Table 2. Groups A and C had a higher incidence of headache, dizziness, and orthostatic hypotension compared to Group B. There was a significant difference among the groups regarding the incidence of headache (P < 0.05). Furthermore, the incidence of anejaculation was significantly higher in the silodosin (73.9%) and combination (84%) groups compared to the mirabegron group (P < 0.05), as shown in Table 2.

**Table 2:** Treatment outcomes

Variables	Group A No. = 35	Group B No. = 35	Group C No. = 35	*P value* A vs. B	A vs. C	B vs. C
Stone expulsion rate	20 (57.1%)	18 (51.4%)	33 (94.3%)	0.745	0.04	0.004
Stone expulsion time (days) mean ± SD	14 ± 2.3	11 ± 3.1	7 ± 2.2	0.465	0.001	0.04
Renal colic episodes (no.)	1.6 ± 1.1	0.8 ± 0.06	0.6 ± 0.2	0.001	0.001	0.001
Extra analgesic ampoules (no.)	3.6 ± 2.8	1.4 ± 1.3	0	0.04	0.001	0.001
Drug unwanted effects. (%)						
Orthostatic hypotension	11 (31.4%)	0	11 (31.4%)	0.001	–	0.001
Dizziness	5 (14.3%)	1 (2.9%)	6 (17.1%)	0.001	0.765	0.04
Headache	12 (34.3%)	3 (8.6%)	10 (28.6%)	0.04	0.567	0.001
*Anejaculation	17/23 (73.9%)	4/22 (18.2%)	21/25 (84%)	0.04	0.435	0.001
Post treatment creatinine	1.12	1.07	1.11	0.821	0.903	0.835

*In males

## Discussion

Spontaneous stone expulsion occurs in about 50% of patients, but problems like ureteral colic, UTIs, and hydronephrosis might occur. The use of adjuvant drugs in MET for distal ureteral stones has shown increased stone clearance rates and decreased pain and complications [8].

Highly selective alpha-1A-adrenoceptor blockers, such as silodosin, have emerged as a way to reduce cardiovascular side effects while maintaining effectiveness in the urinary tract. However, these drugs can still cause unwanted effects such as postural hypotension, anejaculation, and dizziness [9].

To address the need for therapeutic agents with different mechanisms of action and fewer side effects, the study explores the use of beta-3 adrenoceptor agonists for ureteral dilation [10].

Real-time quantitative PCR studies have shown that the dilated distal ureter has fewer beta-3 adrenoceptors compared to a healthy part, suggesting the importance of these receptors in ureteral dilation. [11] Based on this data, the study was designed as a prospective, randomized trial to evaluate the efficacy and safety of tamsulosin, mirabegron, or both as MET for distal ureteral stones.

### Stone expulsion rate

In this study, we show stone expulsion rates in different treatment groups. In the silodosin group (Group A), stone expulsion was reported in 20 out of 35 patients (57.1%), while in Group B, which received mirabegron, it was observed in 18 out of 35 patients (51.4%). In Group C, which received a combination of both medications, stone expulsion was reported in 33 out of 35 patients (94.3%). The stone expulsion rate was significantly higher in Group C compared to Groups A and B, with P-values of 0.04 and 0.004, respectively. However, there was no statistically significant difference in stone expulsion rates between Group A and Group B, with a P-value of 0.745.

Previous studies have reported stone expulsion rates for silodosin ranging from 66 to 84% for stone sizes less than 10 mm, which is higher than the rates observed with placebo, naftopidil, or tamsulosin [12, 13]. Our finding of a 57.1% stone expulsion rate in the silodosin group for patients with stone sizes between 6 and 9 mm is consistent with the results reported by Itoh et al. [14].

Solakhan et al. conducted a study on patients with distal intramural ureter stones and found that mirabegron resulted in a stone expulsion rate of 73.5%. They observed a significant difference in stone sizes less than 10 mm between the mirabegron group and the control group. However, contrary to our findings, Tang et al. and Solakhan et al. [15, 16] did not find significant effects when combining mirabegron with tamsulosin or diclofenac for stones larger than 5 mm. This divergence in results might be attributed to the use of different drug combinations involving mirabegron in those studies.

Bayar et al. conducted a randomized multicenter research study to evaluate the effectiveness of mirabegron and silodosin in patients with stones ranging from 4 to 10 mm. They reported similar rates of stone expulsion across all groups since they established a control group instead of a combination group, which contrasts with our study [17].

In our current study, Group C, which received combination therapy, exhibited a significantly higher stone expulsion rate of 94.3%. This outcome can be attributed to the administration of two drugs with distinct mechanisms of action.

### Stone expulsion time

The average expulsion time (standard deviation) for Groups A, B, and C was 14 ± 2.3 days, 11 ± 3.1 days, and 7 ± 2.2 days, respectively. The combination group (C) had a significantly shorter stone expulsion time compared to the silodosin group (A) and mirabegron group (B) (p = 0.001 and p = 0.04, respectively). Solakhan et al. reported mean stone expulsion times of 7.64 days and 9.2 days for the mirabegron group in distal ureteric stone in different studies. Consistent with our findings, some trials reported stone expulsion times ranging from 10.27 to 14.8 days in the silodosin group for distal ureteric stones [13, 14, 18]. However, other studies reported shorter expulsion times, ranging from 8.09 to 9.4 days [19, 20].

The numbers of renal colic episodes and the need for analgesics.

Ureteric colic occurs when there is increased pressure within the ureter proximal to the site of obstruction. Alpha-adrenergic receptor (AR) antagonists block the C fibers responsible for mediating ureteric colic [21].

Several in vivo studies in animals have demonstrated the relaxant effect of beta-3 agonists on the ureter, leading to a significant decrease in intraluminal pressure. Mirabegron, a beta-3 agonist, relaxes ureteric musculature and dilates the ureteral lumen by stimulating beta-3 adrenoreceptors. This mechanism of action makes mirabegron a potentially effective and safe alternative for medical expulsive therapy (MET), which operates through different pathways [22].

The mirabegron group (B) had a lower frequency of renal colic episodes in comparison to the silodosin group (A) (0.8 ± 0.06 vs. 1.6 ± 1.1, p = 0.001). The combination group (C) had an even lower frequency (0.6 ± 0.2), and fewer analgesics were required (P = 0.001) Recent clinical trials have identified a notable distinction between the mirabegron group and control groups regarding the occurrence of renal colic episodes and the requirement for analgesics in patients with distal ureter stones [15, 17]. Kumar et al. documented an average of 0.8 pain episodes in the silodosin group [18].Substantial evidence suggests that the administration of mirabegron, in combination with other alpha-adrenoreceptor antagonists, for the treatment of distally located ureteral stones is associated with improved stone-free rates (SFR), reduced stone expulsion intervals, and fewer colic attacks [23–26].

### Adverse effects

In our study, no serious adverse effects were observed because both drugs are safe and well tolerated. Anejaculation occurred in 17 out of 23 patients (73.9%) in group A and 21 out of 25 patients (84%) in group C, but no patient discontinued the treatment. The condition was reversible and resolved quickly after stopping the treatment.

Blood pressure and pulse rate did not significantly alter in our study patients. Our findings are supported by a review of the literature, which reveals that a 50 mg dose of mirabegron is not connected to changes in blood pressure or heart rate [27].

Silodosin, which is a highly selective α1A-AR blocker, demonstrates a better stone expulsion rate (57.1%) compared to mirabegron (51.4%). However, mirabegron has the advantage of reducing the stone expulsion time (11 ± 3 vs. 14 ± 2.3 days), numbers of renal colic, and the analgesia requirements. It also has a favorable safety profile with low complications. Therefore, mirabegron shows promise as a medical expulsive therapy (MET) agent for patients with distal ureteric stones.

When silodosin and mirabegron are combined, there is an even higher stone-free rate (94.3%), shorter expulsion times (7 ± 2.2 days), and a reduction in episodes of renal colic (0.6 ± 0.2). This combination therapy offers the advantage of fewer colicky episodes as well. Therefore, for distal ureteric stones with a diameter of ≤ 10 mm, it is recommended to consider the addition of silodosin as a therapy, and mirabegron can be administered to help reduce numbers of renal colic and the analgesia requirements.

### Limitations of the study

The limitations of our study are that non-contrast CT was not done in the follow-up period to assess the stone-free rate due to financial necessity. Silodosin is a labeled medication for lower ureteric stone, but mirabegron is not yet labeled for the treatment of lower ureteric stone. Increases the cost of treatment due to the combination of drugs. Additionally, the small sample size and single-center work suggest the need for larger studies to be conducted.

## Conclusions

The current study concluded that silodosin and mirabegron are effective therapies for the expulsion of lower ureteric stones, Overall, the combination of therapies for lower ureteric stones offers increased stone expulsion rates, shorter expulsion times, better pain control, and a favorable safety profile. This approach represents an effective and comprehensive strategy for managing lower ureteric stones and improving patient outcomes. without any serious adverse effects. Additional research involving a large sample size and multiple centers is necessary.
